# Machine learning techniques to characterize functional traits of plankton from image data

**DOI:** 10.1002/lno.12101

**Published:** 2022-06-30

**Authors:** Eric C. Orenstein, Sakina‐Dorothée Ayata, Frédéric Maps, Érica C. Becker, Fabio Benedetti, Tristan Biard, Thibault de Garidel‐Thoron, Jeffrey S. Ellen, Filippo Ferrario, Sarah L. C. Giering, Tamar Guy‐Haim, Laura Hoebeke, Morten Hvitfeldt Iversen, Thomas Kiørboe, Jean‐François Lalonde, Arancha Lana, Martin Laviale, Fabien Lombard, Tom Lorimer, Séverine Martini, Albin Meyer, Klas Ove Möller, Barbara Niehoff, Mark D. Ohman, Cédric Pradalier, Jean‐Baptiste Romagnan, Simon‐Martin Schröder, Virginie Sonnet, Heidi M. Sosik, Lars S. Stemmann, Michiel Stock, Tuba Terbiyik‐Kurt, Nerea Valcárcel‐Pérez, Laure Vilgrain, Guillaume Wacquet, Anya M. Waite, Jean‐Olivier Irisson

**Affiliations:** ^1^ Sorbonne Université, CNRS, Laboratoire d'Océanographie de Villefranche Villefranche‐sur‐Mer France; ^2^ Sorbonne Université, Laboratoire d'Océanographie et du Climat, Institut Pierre Simon Laplace (LOCEAN‐IPSL, SU/CNRS/IRD/MNHN) Paris France; ^3^ Département de Biologie Université Laval Québec Canada; ^4^ Takuvik Joint International Laboratory Université Laval‐CNRS (UMI 3376), Québec‐Océan, Université Laval Québec Canada; ^5^ Universidade Federal de Santa Catarina (UFSC) Florianópolis Santa Catarina Brazil; ^6^ ETH Zürich Institute of Biogeochemistry and Pollutant Dynamics Zürich Switzerland; ^7^ Laboratoire d'Océanologie et de Géosciences Université du Littoral Côte d'Opale, Université de Lille, CNRS, UMR 8187 Wimereux France; ^8^ Aix‐Marseille Université, CNRS, IRD, Coll. de France, INRAE, CEREGE Aix en Provence France; ^9^ Scripps Institution of Oceanography, University of California San Diego La Jolla California; ^10^ Department of Fisheries and Oceans Maurice Lamontagne Institute Mont‐Joli Québec Canada; ^11^ Ocean Biogeosciences National Oceanography Centre Southampton UK; ^12^ National Institute of Oceanography, Israel Oceanographic and Limnological Research Haifa Israel; ^13^ KERMIT, Department of Data Analysis and Mathematical Modelling Ghent University Ghent Belgium; ^14^ Alfred Wegener Institute for Polar and Marine Research Bremerhaven Germany; ^15^ Centre for Ocean Life, DTU‐Aqua Technical University of Denmark Kongens Lyngby Denmark; ^16^ Laboratoire de Vision et Systèmes Numériques Université Laval Québec City Québec Canada; ^17^ Institut Mediterrani d'Estudis Avançats (IMEDEA, UIB‐CSIC) Balearic Islands Spain; ^18^ Université de Lorraine, CNRS, LIEC Metz France; ^19^ Eawag Dübendorf Switzerland; ^20^ Aix Marseille University, Université de Toulon, CNRS, IRD, MIO UM Marseille France; ^21^ Helmholtz‐Zentrum Hereon Institute of Carbon Cycle Geesthacht Germany; ^22^ GeorgiaTech Lorraine CNRS IRL GT‐CNRS Metz France; ^23^ IFREMER, Centre Atlantique, Laboratoire Ecologie et Modèles pour l'Halieutique (EMH) Unité HALGO, UMR DECOD Nantes France; ^24^ Kiel University Kiel Germany; ^25^ Graduate School of Oceanography University of Rhode Island Narragansett Rhode Island; ^26^ Woods Hole Oceanographic Institution Woods Hole Massachusetts; ^27^ Department of Basic Sciences Cukurova University, Faculty of Fisheries Adana Turkey; ^28^ Centro Oceanográfico de Málaga, IEO, CSIC Fuengirola Spain; ^29^ IFREMER, Laboratoire Environnement Ressources Boulogne‐sur‐Mer France; ^30^ Ocean Frontier Institute, Dalhousie University Halifax Nova Scotia Canada

## Abstract

Plankton imaging systems supported by automated classification and analysis have improved ecologists' ability to observe aquatic ecosystems. Today, we are on the cusp of reliably tracking plankton populations with a suite of lab‐based and in situ tools, collecting imaging data at unprecedentedly fine spatial and temporal scales. But these data have potential well beyond examining the abundances of different taxa; the individual images themselves contain a wealth of information on functional traits. Here, we outline traits that could be measured from image data, suggest machine learning and computer vision approaches to extract functional trait information from the images, and discuss promising avenues for novel studies. The approaches we discuss are data agnostic and are broadly applicable to imagery of other aquatic or terrestrial organisms.

Over the past 40 yr, in situ and laboratory‐based plankton imaging systems have developed rapidly and proven capable of describing the distribution of organisms on unprecedented spatiotemporal scales, from microns to ocean basins and from seconds to decades (Ortner et al. 1979; Davis et al. 1992; Olson and Sosik 2007; Gorsky et al. 2010; Picheral et al. 2010; Sieracki et al. 2010; Ohman et al. 2018). There is a huge variety of plankton imaging devices with a range of technical and ecological applications: living or preserved samples; in the laboratory or in the field; snapshots or continuous video; color or grayscale; coherent or incoherent illumination (Benfield et al. 2007; Lombard et al. 2019). Each tool is limited to a specific portion of the plankton size spectrum and taxonomic tree by illumination and magnification constraints. Combining such modalities would allow aquatic ecologists to study the full‐size spectrum of plankton communities and provide a holistic view of the planktonic ecosystem (Stemmann and Boss 2012; Romagnan et al. 2015; Lombard et al. 2019). These imaging systems enable the simultaneous estimation of the taxonomic structure of a community and measurement of morphological characteristics of individual organisms. The amount of recorded data has increased immensely, both in quantity and in quality, due to improvements in instrumentation—higher pixel density sensors, faster bus speeds, and more powerful embedded computers—and developments in image post‐processing. Moreover, using imaging techniques on preserved net‐ or bottle‐based plankton samples has given scientists the opportunity to revisit historic archives (García‐Comas et al. 2011; Peacock et al. 2014).

The principal objective of plankton imaging has been to extract quantitative, population‐level information through taxonomic recognition of individual objects. Manual annotation of image data remains a nontrivial task that requires expert knowledge and lots of time, even with semiautomated recognition (Culverhouse et al. 2003, 2014; Grosjean et al. 2004). Human labeling has remained the major bottleneck limiting scientists' ability to analyze image data or use it for monitoring purposes (MacLeod et al. 2010). Scientists have started to look toward supervised machine learning (ML) methods—algorithms that learn to classify new data from a set of human‐generated training examples—to expedite classification efforts (Irisson et al. 2022). Until the 2010s, such methods typically involved extracting hand‐engineered numerical features from a curated set of training images followed by tuning an ensemble or margin‐based classifier (Simpson et al. 1991; Blaschko et al. 2005; Sosik and Olson 2007; Gorsky et al. 2010). Computers were programmed to measure shape attributes—such as area, eccentricity, or aspect ratio—and texture metrics—like variance in gray levels—from the images. These routines were generally written for data collected by a specific imaging system and required manual parameter tuning to ensure accurate measurements. Recently, aquatic ecologists have started applying popular deep‐learning techniques that have surpassed previous state‐of‐the‐art classification performance on both benchmark and plankton specific datasets (LeCun et al. 2015; González et al. 2017; Orenstein and Beijbom 2017; Luo et al. 2018; Ellen et al. 2019). Deep methods learn representations directly from images, obviating the need for human defined shape and texture attributes, but require an enormous amount of annotated training data to appropriately tune (Bengio et al. 2013; Sun et al. 2017).

Functional trait‐based approaches (FTBAs) describing plankton ecosystems have been developed in parallel with advances in imaging systems (Litchman and Klausmeier 2008; Litchman et al. 2013; Kiørboe et al. 2018; Martini et al. 2021). Rather than considering the taxonomic identity of an organism, FTBAs instead characterize them by their specific combination of functional traits—characteristics of individual organisms that impact their fitness via resource acquisition, growth, reproduction, and survival (Violle et al. 2007). Trait information transcends taxa and may have several advantages over classical taxonomic information: (1) organisms are distributed in the environment mainly according to their traits, not their taxonomic classification; (2) ecosystem functions depend on the traits of constituent organisms, not their taxonomic composition; and (3) while aquatic ecosystems are inhabited by a myriad of species (e.g., hundreds of thousands of eukaryotic taxa in the upper ocean; de Vargas et al. 2015), key taxa‐transcending traits are few (Martini et al. 2021). Trait‐based descriptions therefore have the potential to encapsulate the complexity of aquatic ecosystems in a concise number of metrics (Kiørboe et al. 2018).

The functional trait‐based perspective suggests that obtaining information on the characteristics of individual organisms directly from images is an efficient alternative to sorting the data taxonomically (Martini et al. 2021). Indeed, efforts have already been made to reanalyze hand‐engineered image features originally drawn for classification purposes. This approach has revealed cryptic patterns in plankton communities, such as the morphological characteristics of particulate matter in the global ocean and the feeding habits of marine copepods along an Arctic sea‐ice gradient (Trudnowska et al. 2021; Vilgrain et al. 2021). Such studies are just beginning to combine high‐throughput imaging systems and automated analysis techniques for trait‐based studies. We believe there is much more to be done and learned by leveraging a suite of powerful new, deep neural network‐based, ML tools in the context of functional trait‐based ecology.

This paper aims to highlight how automated processing techniques might be applied to gain direct access to plankton functional traits from individual images and, eventually, enrich our understanding of pelagic ecosystems. First, we detail traits that lend themselves to observation in images, regardless of how the image has been acquired (in situ or in the lab, high throughput or piecewise acquisition, etc.). We distinguish between traits that have a clear visual signature and can be *measured* from the image itself and traits that are *inferred* from the image, taking into account additional assumptions or contextual variables. The second section is dedicated to ML methods that could be used to estimate functional traits from images with a focus on new deep neural network procedures. These techniques rely on human annotations to learn general representations of desired traits that are perhaps inaccessible via an algorithm designed to make a particular measurement. We encourage ecologists to adopt an “evaluation‐first” design paradigm to inform data annotation and algorithm selection. We conclude with several forward‐looking suggestions regarding potential statistical analyses of the traits collected by ML algorithms, future instrument design to collect functional trait data, and extensions of these methods to other organisms.

## Plankton traits from images

**Fig. 1:**
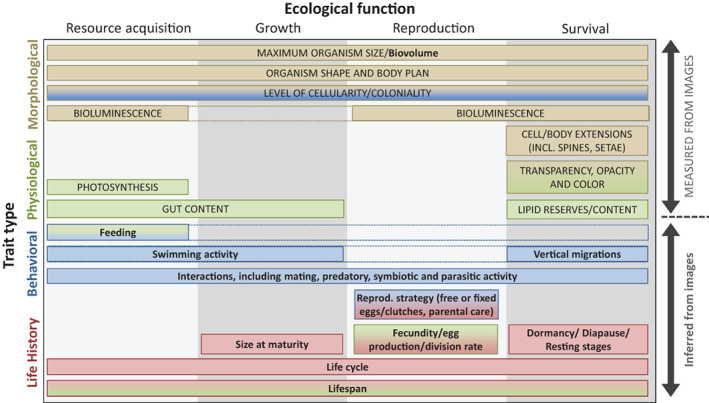
Plankton functional traits that can be estimated from images, following the unified typology of Martini et al. (2021). Trait types along the *y*‐axis follow the order of the “Plankton traits from images” section. Measured traits, ones that can be quantified solely from images, are in capital letters. Inferred traits, which require additional information beyond raw pixels, are written in bold text.

Imaging of planktonic organisms enables studies that track whole communities while collecting individual‐level metrics such as functional traits (Fig. 1). In situ systems, in particular, can capture images of undisturbed creatures that reveal their pose, position, behavioral responses, and local interactions between organisms (Ohman 2019; Vilgrain et al. 2021).

For the rest of this paper, we primarily discuss images that are already segmented into regions of interest (ROIs). For most plankton imaging systems, it is relatively easy to remove targets from full frame images since they are sparsely distributed against uniform background intensities (Gorsky et al. 2010; Orenstein et al. 2020). Plankton imaging systems usually select ROIs by distinguishing foreground pixels from the background without regard to the actual nature of the object. Therefore, the instrument does not discriminate between living creatures and particles such as marine snow (fecal pellets and other organic detritus). At any time and depth in pelagic systems, abundance, and biomass of marine snow dominate and thus form the bulk of collected image data (Alldredge and Silver 1988; Stemmann and Boss 2012; Ohman et al. 2018; Trudnowska et al. 2021). Analysis of ROIs of marine snow is an important area of active research, but is not the focus of this work. In this section, we will focus on individual organisms' traits, following the typology presented in Fig. 1, and discuss how these traits could be measured or inferred from images.

## Measured traits

### Size: The “master trait”

**Fig. 2:**
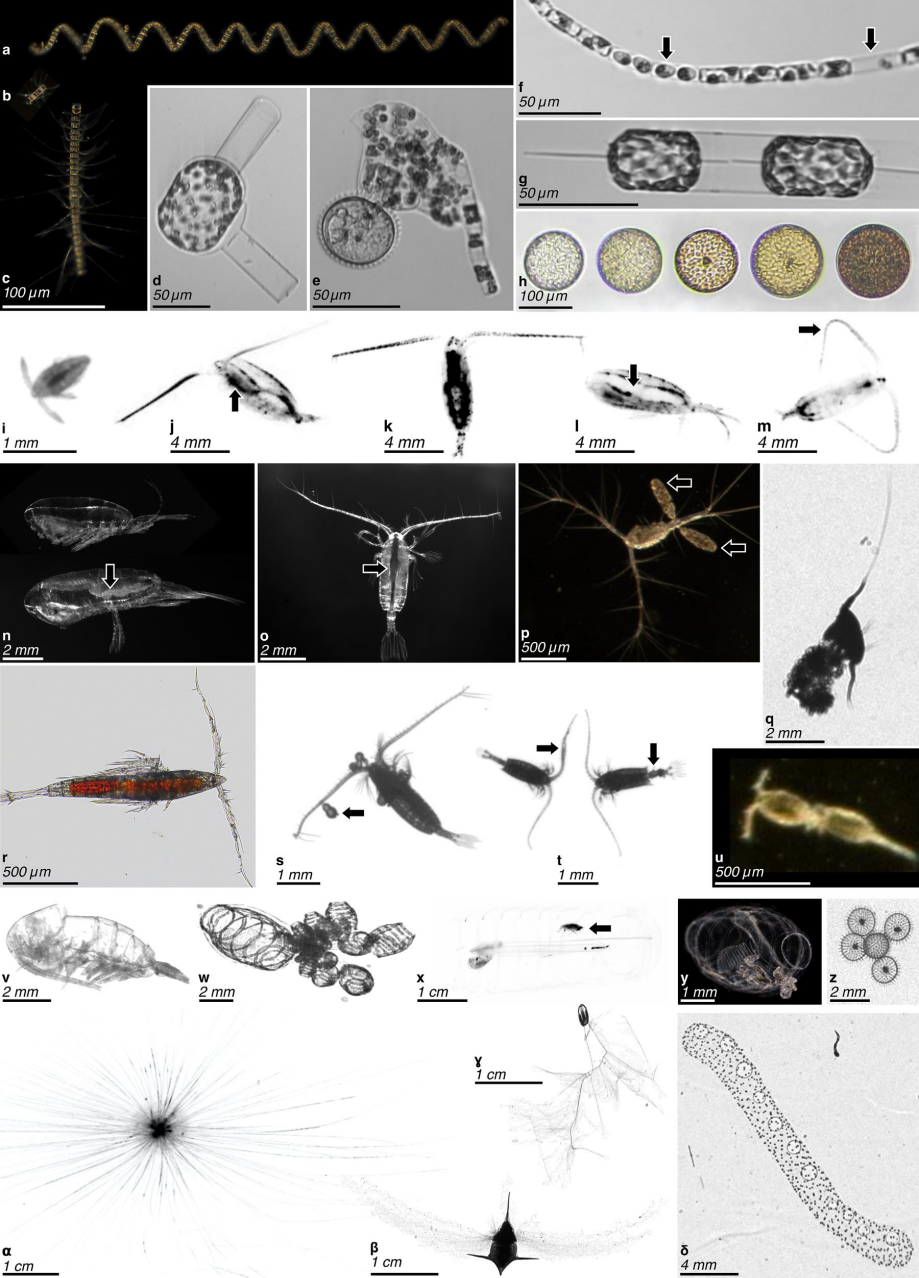
Example of plankton images on which traits can be identified. (**a**–**h**) Diatoms, (**i**–**v**) copepods, (**w**–**δ**) other taxa. (**a**–**c**) Chains of *Chaetoceros* spp. of different sizes (Scripps Pier Cam [SPC]); note the long spines on (**c**). (**d**) Sexual stage of *Guinardia flaccida* (Imaging FlowCytobot [IFCB]). (**e**) Dinoflagellate consuming a diatom chain (*Guinardia delicatula*) by external digestion in a feeding veil (pallium) (IFCB). (**f**) *Guinardia delicatula* infected with parasite (first arrow) or as an empty frustule (second arrow) [IFCB]. (**g**) *Ditylum brightwellii* cell dividing (IFCB). (**h**) Coscinodiscophycidae (centric diatoms) containing various amounts of pigments (Planktoscope). (**i**) Nauplius stage of a crustacean (ZooScan), (**j**–**m**) calanoid copepods (Underwater Vision Profiler 5), note the full gut (arrow) and active posture with antennae deployed on (**j**), the pigmented (dark) body parts on (**j**–**l**), the lipid sac (arrow) and resting posture, with antennae along the body on (**l**), and the curved antennae (arrow) associated with a jump of the copepod on (**m**). (**n**) Immature (top) and mature (bottom, with visible oocytes—arrow) female of *Calanus hyperboreus* (Lightframe On‐sight Key species Investigation [LOKI]). (**o**) *Gaetanus brevispinus* displaying many sensory setae on its antennae and a well visible gut (arrow) (LOKI). (**p**) Another copepod with well visible setae and two egg sacs (arrows) (SPC). (**q**) Copepod associated with (possibly feeding on) a marine snow particle (ZooGlider). (**r**) *Microsetella* sp. displaying many spines and intense coloration, likely from its gut content (Planktoscope). (**s**) Calanoid copepod with parasite dinoflagellates (arrow) (ZooCAM). (**t**) Male (with geniculate antennae—arrow, left) and female (with bulging genital segment—arrow, right) of *Centropages* sp. (ZooCAM). (**u**) Oncaea mating [SPC]. (**v**) Empty copepod carcass or molt (ZooScan). (**w**) Doliolid budding (ISIIS). (**x**) Salp with an amphipod inside (arrow) (UVP). (**y**) Transparent Doliolid (SPC). (**z**) A few solitary Rhizaria, family Aulospheridae (ZooGlider), to be contrasted with (**δ**). (**α**) Foraminifera with long cell extensions (UVP). (**β**) Pteropod (dark) with part of its mucus net deployed (gray). (**ɣ**) Ctenophore, family Mertensiidae, with very long fishing tentacles deployed (ISIIS). (**δ**) A colonial Rhizaria, order Collodaria (ZooGlider).

Cell or body size is a functional trait that can be readily measured from a calibrated image (Fig. 2a–c). Indeed, size is the only directly measured trait that has been consistently analyzed from image data (Picheral et al. 2010; Giering et al. 2020). Size transcends the scales of organization of biological systems, from the individual to the whole ecosystem through allometric relationships involving metabolism, development, feeding, and mobility (Fig. 1; Platt and Denman 1977, Hansen et al. 1994, Litchman et al. 2013, Blanchard et al. 2017). In aquatic ecology, the size structure of the whole community of organisms has been recognized as a property explaining trophic network organization and functioning since the early 1970s (Sheldon et al. 1972; Sieburth et al. 1978). It is hence often referred to as a “master trait.”

Size should be understood in all its dimensions, not only in terms of length (L^1^) but also surface (L^2^) and volume (L^3^) or, equivalently, individual biomass. The major axis length is a first‐order estimate of size measurable from images. Surface can be estimated from the cross‐sectional area an object occupies in the image plane. Volume can likewise be derived from the particle's 2D projection under a set of assumptions (Moberg and Sosik 2012). These metrics, computed either through length measurements or equivalent spherical diameter (ESD), are the standard approach to estimating size spectra from sets of individual plankton image data. The measurements, however, must be calibrated between systems and deployments since they can vary due to foreshortening as a function of the object's orientation relative to the image plane, the imaging system itself, or the image processing pipeline (Giering et al. 2020).

Beyond size, other morphological traits have not been studied as extensively since plankton were historically sampled with nets or bottles. While effective for taxonomic identification, these extractive techniques can damage or destroy fragile aquatic organisms, hampering efforts to collect information about the relationship between the individual's morphology^1^ and its environment (Remsen et al. 2004; Whitmore et al. 2019). In situ imaging devices can, however, measure these intrinsic and contextual elements without altering an individual's expression of its morphological traits (Ellen et al. 2019).

### Organism shape and body plan

The shape of an individual organism is intimately linked to its biological function and the way it interacts with the physical environment (Kiørboe 2011b; Hirst 2012; Ryabov et al. 2021). The particular hydrodynamic environment an organism lives in imposes strict constraints on body plans. A single organism can experience several orders of magnitude of Reynolds numbers while growing from an egg to adulthood, from a “sticky” laminar flow to the turbulent flow humans experience while swimming. As a result, it usually develops different locomotion strategies throughout its ontogeny. Copepods, for example, develop from spherical nauplius larvae that generate propulsion with appendages surrounding their body, to torpedo‐shaped copepodite stages equipped with appendages allowing them to hover or jump by hundreds of body lengths per second to escape threats detected via flow disturbances (Fig. 2i,j; Kiørboe et al. 2010).

Unicellular organisms, such as diatoms or rhizarians, can likewise alter the shape of the biological unit they are forming, for example, by adopting colonial strategies and forming colonies or chains (Fig. 2a–c,z,*δ*; Biard and Ohman 2020, Kenitz et al. 2020). This strategy can be related to the need to (1) counter predation by inflating size, (2) modify the surface‐to‐volume ratio to increase resource acquisition, and (3) modify the drag coefficient to control settling velocities, or any combination thereof (Nielsen 2006; Du Clos et al. 2021). Such strategies can impact global‐scale biogeochemical cycles by altering the size‐spectrum of the planktonic community and the balance between the productive surface layer and the interior of the ocean.

### Cell/body extensions

Many planktonic organisms bear spines, stout setae, and other defensive structures that inhibit consumption by predators. Such structures (elongated spines on diatoms—Fig. 2c, stout caudal setae of some harpacticoid copepods, etc.) are optically resolvable and likely to confer a reduction in predation rate.

In other plankton, body extensions are involved in feeding. In situ imaging has revealed that the volume of water filtered by these aquatic organisms may be markedly different than expected from net‐collected individuals (Ohman 2019). For example, recent results from mesopelagic foraminifera have shown that the complete resolution of calcite spines and rhizopods can increase the surface area of the foraging apparatus by 100–1000 times relative to what was expected from the hard test alone (Fig. 2α; Gaskell et al. 2019). In situ images of several types of tentacle‐bearing predators, including cnidaria and ctenophores, have also shown dramatically larger surface areas when the full extent of natural swimming and hunting postures is taken into account (Whitmore et al. 2019). Many pteropods and appendicularians use large and elaborate mucus extensions that filter vast quantities of water (Fig. 2β; Alldredge 1981, Burridge et al. 2017). These fragile gelatinous structures have broad implications for the biological carbon pump by increasing particle flux to the deep ocean (Lombard and Kiørboe 2010; Katija et al. 2017). The new information revealed by imagery has forced a re‐evaluation of the classical approach to aquatic trophic network dynamics and caused an update of some allometric relationships between body size and filtering rates and even, in some instances, prey size (Conley et al. 2018).

Finally, many organisms (especially crustaceans) have a variety of mechanosensory and chemosensory setae that are crucial for prey and threat detection and which can be resolved by imaging systems (Fig. 2o,p). For example, prey–predator interactions have been observed for the marine copepod *Oithona plumifera* by analyzing frames from experimental video recordings (Jiang and Paffenhöfer 2008). This advancement could have important consequences for trophic network analyses and our understanding of matter and energy flows, since the sensory abilities of zooplanktonic organisms are tightly linked to their feeding strategies (Kiørboe 2011a).

### Bioluminescence

Bioluminescence is the emission of light by living organisms resulting from a chemical reaction. It is a ubiquitous trait known to exist in about three quarters of planktonic organisms down to 4000 m (Martini and Haddock 2017). Light emitted by organisms can differ in terms of pattern and intensity: luminous spots on a body‐part, repeated pulses of bright light by gelatinous organisms, diffuse sources of luminous excreted material, or chains of luminescent structures (Priede et al. 2006). Regardless of the specific manifestation, bioluminescence is strongly related to intraspecific communication—such as mate finding—or predator–prey interactions (Haddock et al. 2010). Until recently, most of the biological functions attributed to this trait have been inferred from the organism ecology, morphology, and the characteristics of the bioluminescent emission (wavelength, intensity, patterns, and chemistry).

Bioluminescent signals are easy to spot, either by in situ observations or in the laboratory after collecting organisms, and can be spontaneous or mechanically stimulated. Hence, it remains difficult to assess whether measurements reflect natural behavior or the organism's response to stimuli associated with the imaging method. Recent deployments of high sensitivity cameras on remotely operated vehicles have collected images of bioluminescence in situ. In the near future, we expect to see such tools used for consistent measurements of this trait, especially if we can accurately assess the level of disturbance created by the sampling devices (Ohman et al. 2018). In the meantime, signal analysis of bioluminescence intensity over time is a promising approach to better understand individual signals as well as community changes over time (Cronin et al. 2016; Messié et al. 2019).

### Transparency, opacity, and color

Tissue transparency is an important characteristic of the body plan of metazoans, including cnidarians, ctenophores, chaetognaths, and pelagic tunicates (contrast Fig. 2k,t with Fig. 2w,x,y). The combination of nearly transparent tissue with high water content allows organisms to be large, often with reduced metabolic requirements and reduced susceptibility to visual predators (Acuña et al. 2011). Opacity can likewise offer crucial information about many zooplanktonic organisms since body structures, such as gonads (Fig. 2k,n) and guts (Fig. 2j,o), can have variable content and appearances in the image (Vilgrain et al. 2021). Gut fluorescence, for example, has been used for several decades to estimate grazing rates on phytoplankton (Pasternak 1994), and can reveal whether feeding was recent or not (Sourisseau et al. 2008). Other pigments may be linked to physiological functions, such as carotenoids which offer oxidative stress protection to marine and freshwater copepods (Hylander et al. 2015; Schneider et al. 2016).

For unicellular organisms, pigmentation has been measured for decades from individual cells as a monochromatic or multispectral fluorescence signal (Olson et al. 1991, 2003) and can now be acquired alongside images (Olson and Sosik 2007; Sieracki et al. 2010). Individual pictures can yield the identification and quantification of intracellular structures, such as chloroplasts (Fig. 2h) of photosynthetic symbionts, epibionts, or parasites (Fig. 2f). Pigments can also be measured either in their cell of origin (Fig. 2h), or inside the vacuoles or the gut of a consumer (Fig. 2r). Thus, a combination of image measurements may be required to assess whether the pigmentation actually originates from the individual observed, especially in the complex case of mixotrophic organisms (Flynn et al. 2013; Lauffer et al. 2015).

### Photosynthesis

Among the main light‐acquisition traits (Litchman and Klausmeier 2008; Kiørboe et al. 2018; Martini et al. 2021), the most common can be estimated at the individual level from images using microscope‐based saturating‐flash fluorescence measurements (e.g., quantum yield of photochemistry, photosynthesis‐irradiance curves related parameters, nonphotochemical quenching, and functional absorption cross section). This has been performed in natural phytoplankton populations (Olson et al. 1996; Villareal 2004; Dijkman and Kromkamp 2006). Recent developments in hyperspectral imaging combined with optical microscopy will allow estimation of pigment content (Fig. 2h) and spectral absorption properties (Méléder et al. 2013) at the individual scale (Xu et al. 2020).

### Lipid reserves

Lipid allocation is a key component of energy reserves and buoyancy for a variety of organisms. In organisms that have adapted to conditions of intermittent resources, reserves can be found in dedicated internal structures that could be measured directly from images: vacuoles in unicellular plankton, lipid droplets in both unicellular and metazoan organisms, and lipid sacs in zooplankton (Fig. 2l) thriving in extreme seasonal conditions (e.g., upwelling along eastern boundary currents and monsoon or polar oceans). Lipids can represent up to 80% of body volume in polar copepods, for example, and contribute actively to parts of the global carbon cycle through the *lipid pump* (Jónasdóttir et al. 2015). Imaging can be used to visually estimate these lipid reserves in copepods using geometric approaches akin to measuring whole body volume and biomass (Schmid et al. 2018). Lipid droplets in individual phytoplankton cells can be quantified using holotomographic imaging devices (Jung et al. 2018), although this technique is not feasible yet for in situ measurement.

## Inferred traits

The functional traits discussed so far can be measured directly from images. Many others cannot but could rather be estimated by combining visible features and environmental context metrics obtained from the sampling device metadata (temperature, light level, etc.).

### Biomass and biovolume

Biovolume and biomass, as opposed to length and shape, can be estimated from an organism or cell measured area using empirical relationships specifically derived for a particular imaging instrument and target organism (Hillebrand et al. 1999; Menden‐Deuer and Lessard 2000; Moberg and Sosik 2012). Biomass estimates from biovolume can involve simple conversion factors and ratios that may vary between organisms. For example, a jellyfish and a crustacean of the same ESD have distinct biomass conversion factors (Lehette and Hernández‐León 2009; McConville et al. 2017; Giering et al. 2019). These relationships can be generic for whole groups or calibrated for individual species or development stages (Ikeda et al. 2007). Beyond biomass, carbon and nitrogen content per cell could be estimated for nondiatom marine phytoplankton species whose shape roughly conforms to prolate spheres, as demonstrated by Verity et al. (1992).

### Feeding and metabolic rates

In situ images of relatively transparent aquatic organisms can be used to estimate the volume swept while feeding (Fig. 2α–ɣ), the digestive tract fullness (Fig. 2j,o), and the frequency of actual feeding interactions (Fig. 2e,q). Ingestion rates, gut transit time and maximum feeding rates could subsequently be estimated with concurrent measurements of environmental variables such as temperature and prey concentrations (Wirtz 2013). Metabolic activities, such as respiration and excretion, scale with organism size (Banse 1976; Ikeda et al. 2001), allowing the estimation of, for example, zooplankton community respiration or excretion as the sum of all individual organisms' activities (Ikeda 2014).

### Swimming and activity

Aquatic organisms have evolved diverse locomotive structures with clear morphological signatures like gas vacuoles, flagella, and cilia. An organism's specific motility strategy affects the trade‐off between energy intake through feeding and death by predation (Harvey and Menden‐Deuer 2012). It also indicates if the organism can actively influence its position in the water column and overcome turbulent mixing (Gallager et al. 2004) (Fig. 2m, of a copepod “jumping”). Activity patterns can potentially be inferred from still images based on the position of body and appendages. For example, Vilgrain et al. (2021) suggested that the “body contour complexity” defined by their multivariate analysis of thousands of individual images of Arctic copepods revealed that copepods were hovering and foraging in the marginal ice zone in spring when the phytoplankton bloom occurred (Fig. 2j,k). Under continuous ice cover, individual copepods displayed a posture typical of dormancy (Fig. 2l).

Diel vertical migration (DVM) is a peculiar case of swimming activity that influences the fitness of a range of highly mobile aquatic organisms. Zooplankton evolved this behavior as a trade‐off between maximizing feeding opportunities on the primary producers thriving in the well‐lit surface and minimizing mortality from visual predators (Aksnes and Giske 1990; Möller et al. 2015). Recent results suggest that both the propensity to migrate vertically and the amplitude of DVM varies with the body size of planktonic copepods (Ohman and Romagnan 2016). The accurate characterization of metazoan body size could then be combined with visual indices of activity level to predict the migration behavior of zooplanktonic organisms.

### Interactions

Interactions among organisms or between organisms and particles can be split in two categories: the first involves interactions between distinct entities spread within the water column (e.g., predator and prey; Fig. 2e), while the second occurs at the scale of an individual (e.g., parasitism; Fig. 2f,s,x). In situ imaging systems that record the undisturbed relative position of individuals could be used to reveal traits related to spatial interactions, such as the feeding (Fig. 2e,q), mating (Fig. 2u), parasite infection (Fig. 2f), or predation. When coupled with the appropriate information extraction technique, such data will allow scientists to better understand inter‐ and intraspecific relationships, from intracellular symbiosis and parasitism to planktonic interactions within microhabitats like marine snow or other detrital matter (Peacock et al. 2014; Nishibe et al. 2015).

### Reproduction strategy, fecundity, and division rate

Reproduction strategies form a continuum bounded by *r* (many offspring, no brooding) and *K* (few offspring, brooding) strategies (Jaspers et al. 2018). Taxa with *r*‐strategies tend to have a smaller size at maturity and to produce large clutches—many eggs are released during a single spawning event—while *K*‐strategists tend to produce larger adults and release a few eggs at a time. *K*‐strategists' eggs are also often much larger, provided with substantial lipid reserves, and benefit from parental care (e.g., brooding pouches from amphipods or egg sacs from copepods).

In situ imaging systems, with sufficient pixel resolution, could be used to count free eggs in the water column, individuals within egg sacs (Fig. 2p; Möller et al. 2015) or to estimate the occurrence of mature oocytes in females' gonads (Fig. 2k,n; Niehoff 2007). Daily individual egg production rates could be estimated from these measures—coupled with simultaneous in situ temperature data, the estimated proportion of mature females, and female size at maturity—via known allometric relationships and temperature‐dependent, species‐specific hatching time (Niehoff 2003). For asexually reproducing organisms, new budding individuals could be directly counted on the image (Fig. 2w), and frequency of cell division (Fig. 2g) could be used to infer protist population level division (Campbell et al. 2010; Brosnahan et al. 2015). Monitoring the evolution of taxonomically resolved planktonic cell size in both time and space could provide information on productivity levels of a system (Hofmann et al. 2019).

### Life cycles and lifespan

The majority of plankton are unicellular organisms whose cell morphology is tightly linked to the phase of an individual's development cycle, including sexual stages (Fig. 2d) and dormant stages (Brosnahan et al. 2015). Most multicellular zooplankton species also have morphologically distinct life stages (Fig. 2i,j). Usually, the individual develops from an egg to adulthood through several molting or metamorphosis events. This process requires a significant energetic investment over time, but it ensures that the morphology of growing individuals is well adapted to their changing environment (Sainmont 2014). The appearance of early larval life stages of crustaceans, pteropods, and polychaetes in the water column is indicative of recent reproduction and successful hatching. Images of sufficiently high resolution to identify these larval stages can thus yield the reproductive phase of a local population and contribute to estimates of the ecosystem productivity. Images could also provide further information on individual ages. Zooplankton age is crucial for studying population and community dynamics but is notoriously difficult to determine (Kiørboe et al. 2015). For example, krill continue to molt throughout their lives to accommodate growth. Krill can also undergo “negative growth” and shrink if environmental conditions are unfavorable for growth by relying on some specific strategies: the use of lipid stores, gonads resorption, etc. In such cases, age cannot be determined from an image captured during an individual development stage, body size, or gonad development. We are not aware of any attempt to accurately estimate a numerical value of the age of individual zooplankton directly from in situ images. It is unlikely that this could ever be achieved, except for very peculiar cases where aging would somehow “scar” an individual in a typical and predictable way.

### Dormancy and resting stages

Phytoplankton resting stages, such as dormant spores, can be inferred from images and used to better estimate carbon fluxes as they play a significant role in carbon export. In the specific context of in situ imagery, diatom spores are an obvious target since they form inside the cell frustule. Live cells and spores can be identified from in situ images of diatoms, which is especially useful with chain‐forming species for providing quantitative estimates of vertical fluxes (Salter et al. 2012; Rembauville et al. 2018). Similarly, zooplankton that accumulate lipid reserves can undergo dormancy (both quiescence and true diapause; Fig. 2l). This trait is associated with a shift in metabolic rate, activity level, habitat, swimming behavior, and survival (Baumgartner and Tarrant 2017). Thus, it has a significant impact on individual fitness, local population dynamics, and the biological carbon pump (Jónasdóttir et al. 2019). Images have been analyzed to characterize dormant and active copepods in the Arctic by collecting individual‐level measurements of stage development, lipid content, and associated depth distribution (Schmid et al. 2018). Such individual measurements can be combined with in situ temperature data to estimate an individual's respiration rates at the depth of dormancy and, eventually, infer the duration of its dormancy phase (Maps et al. 2014).

### Mortality

Living vs. dead organisms can be distinguished both manually and automatically in image data (Fig. 2f,v; Picheral et al. 2017, Reimann et al. 2020). The use of high‐throughput imaging devices deployed in situ offer new opportunities to estimate the spatiotemporal variation in the proportion of dead individuals within natural phyto‐ and zooplankton communities. More generally, the accumulation over time and space of finely resolved trait distributions (e.g., development stages, sizes, and proportion of dead individuals) could significantly increase the usefulness and precision of statistical approaches used to estimate mortality rates and life span based on cohort detection and monitoring (Shaw et al. 2021).

## Computer vision and ML approaches to estimate traits from images

Computer vision and ML techniques offer scalable solutions to automatically quantify functional traits from plankton images (Table 1). Successful deployment of these methods will require careful consideration of the target trait to select the best course of action. Researchers then must collect specialized annotated data, select the candidate algorithms, and evaluate its performance with an appropriate metric.

**Table 1:** Definitions of a few computational terms, highlighting important, but subtle, differences.

Computer vision (CV)	A broad subfield of computer science dedicated to using a computer to interpret images and video sequences.
Machine learning (ML)	A set of statistical approaches that attempt to discern patterns in data, either automatically or based on explicit human instructions.
Supervised ML	ML techniques that teach a computer to recognize patterns using a set of expert‐curated examples, such as annotated images.
Unsupervised ML	ML methods that attempt to group data together without human intervention. Clustering algorithms are a common example. Their performance is often difficult to evaluate.
Training set	A collection of data annotated by human experts for teaching a computer how to interpret information. Building the labeled dataset is the most time‐consuming and critical part of an ML workflow.
Validation set	A separate human labeled dataset used to evaluate a trained system. These data are entirely independent of the training set and should represent conditions the system might encounter in the field. Also referred to as *test* data.
Feature‐based learning	ML algorithms that operate on a reduced, hand‐engineered feature space. Each data point is cast as a vector of measurements and used to tune a set of parameters that dictate how the model works.
Deep neural networks (DNNs)	A type of *representational* algorithm that learns directly from raw data. DNNs layer many mathematical abstractions on top of each other to connect input information to a desired output. Through iterative training, the system learns the most salient features of the input. Modern DNNs often have numerous layers and billions of weights.
Transfer learning	A shortcut for training DNNs by repurposing a network originally trained for a different task.

To this end, we recommend adopting an evaluation‐first design paradigm—where the specific task and quantitative evaluation metric are defined before annotation begins—to motivate the construction of these resources and the necessary workflow (Gupta et al. 2019). Following this principle will encourage scientists to think carefully about the ML method they wish to use, the criteria for success, and therefore what sort of labeled data they need. This output‐centric approach will help ensure human effort is not wasted generating inappropriate labeled data for a given task and minimize barriers to experimentation.

**Fig. 3:**
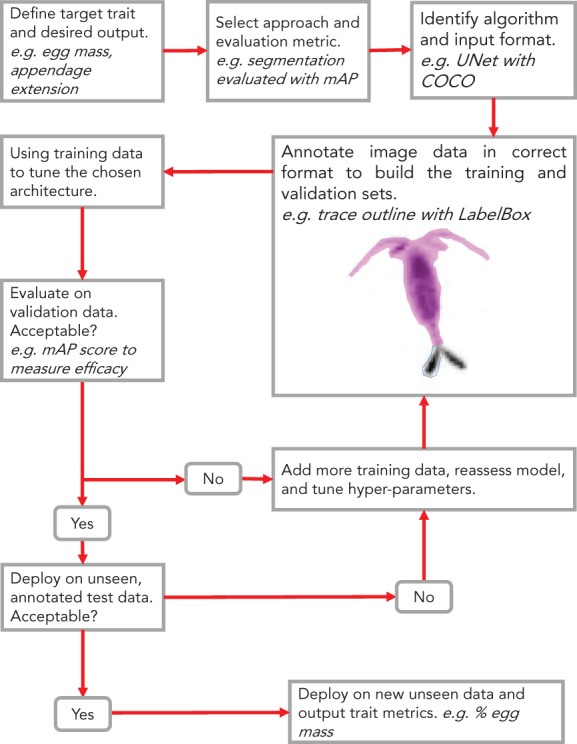
Workflow diagram for a computer vision approach targeting a specific functional trait extracted from plankton image data. In this example, a group decides to target egg‐bearing copepods with a UNet segmentation model. A human annotator selects ovigerous tissue from a copepod image and outputs the mask in the COCO data format. The model is then trained and evaluated using the mAP (Table 2; Supporting Information S1). Note that this workflow is not specific to plankton and could also be used for other types of organisms.

Consider a project seeking to examine copepod egg mass from image data (Fig. 3). The scientists narrowly define the desired output as “the number of egg pixels in an ROI” that can be further analyzed to estimate egg mass. Since the group cares about individual pixels, they determine to experiment with segmentation algorithms that will be evaluated with mean average precision (mAP; Table 2; Supporting Information S1). In order to train and evaluate such models, the team will need to annotate detailed pixel level masks and store them in a compatible format.

**Table 2:** Common evaluation metrics for automated classifiers. See Supporting Information for more detail and description.

Accuracy (acc)	$\frac{t_{p}+t_{n}}{t_{p}+f_{p}+f_{n}+f_{n}}$	The number of true positives in a class returned by an automated classifier over the total number of correct labels in the class.
Precision (*p*)	$\frac{t_{p}}{t_{p}+f_{p}}$	The ratio of the number of correct labels over the total number of labels assigned to that class.
Recall (*r*)	$\frac{t_{p}}{t_{p}+f_{n}}$	The proportion of positive samples in a class that are correctly classified.
F1‐score	$\frac{t_{p}}{t_{p}+\frac{1}{2}\left(f_{p}+f_{n}\right)}$	The harmonic mean of precision and recall that summarizes model performance in a single metric scaling between 0 and 1.
Average precision (AP)	$\frac{1}{n+1}\sum_{\mathrm{r\epsilon}\left\{1/n,2/n,\ldots ,1\right\}}p\left(r\right)$	The average of the precision over different levels of the recall.
Mean average precision (mAP)	$\frac{\sum_{c=1}^{Q}\mathrm{AP}}{Q}$	The mean AP over all classes. It is a summary statistic that describes how a model does over all classes.
Intersection over union (IoU)	$\frac{A\cap B}{A\cup B}$	A∩B represents the area in pixels of the overlap between the region proposed by the computer (*A*) and the ground truth (*B*). A∪B is the total number of pixels contained in both regions. IoU is a metric mostly used for detection and segmentation tasks.

In order to measure functional traits from images, one must design a pipeline to take a project from initial human annotation to deployment on unlabeled data. Here, we discuss how to construct a training dataset, potential computer vision and ML approaches to estimate traits from images, and how to evaluate the trained systems. To our knowledge, none of the deep‐learning methods have been implemented for functional trait analysis of plankton images. We thus suggest reading this section as a guide to broad approaches, not as specific recommendations of algorithms or models.

### Datasets and labeling

The critical step in any supervised ML experiment is the construction of a labeled training set suitable for a given method. For classification tasks, this entails deciding on the appropriate set of classes to consider and annotating the data until there are sufficient labeled samples—typically 100–1000 s per class for deep neural networks—to train the desired model. To examine specific functional traits, an automated analysis system will need to detect and measure specific features. These tasks will require human effort beyond sorting images to generate labeled data that localizes a particular trait, masks relevant pixels, or estimates prevalence. The exact annotation approach depends on the target trait and corresponding computational method.

### Annotation formats

ML frameworks expect data in specific standard formats. To train a classifier, for example, training images are often expected to be sorted into unique folders or accompanied by a numeric list indicating the class names. Object detection, segmentation, and keypoint detection frameworks all likewise require annotations in specific formats for training.

There are two common natural image datasets used for benchmarking new approaches to such tasks: PASCAL Visual Object Classes (VOC)^2^ and Common Objects in COntext (COCO)^3^ (Everingham et al. 2010; Lin et al. 2014). These two databases contain many thousands of images with bounding boxes for object detection and pixel level segmentation labels. Publicly available domain‐specific datasets often adhere to the VOC or COCO formats to facilitate experiments, model evaluation with publicly available tools, and dataset interoperability.

Scientists seeking to fine tune existing architectures trained on these datasets might consider adopting their exact annotation formats. Alternatively, operators could save annotations in whatever format is most convenient and write their own data loader in the selected toolkit. PyTorch, for example, has a generic dataset object that can be modified to accommodate any input and output.

### Annotation tools

There is a broad universe of tools for data annotation, ranging from freeware interfaces to industry‐scale software packages (e.g., LabelBox, RectLabel, and MATLAB Image Labeler). Many of these are open source and can be adapted to include contextual metadata or other parameters. Several tools have been written specifically for in situ marine imagery that facilitate integration of oceanographic metadata (Gomes‐Pereira et al. 2016; Picheral et al. 2017). The choice of annotation software should be dictated by ease of use and the ability to output in standard data formats. Practitioners should invest time researching contemporary options and select a package that is actively being supported. Annotation requirements and format standards will undoubtedly evolve as the technology changes. At the time of writing, researchers should look for services that (1) output data in COCO or VOC format, (2) have an interface for polygon object detection labeling, (3) support superpixel segmentation labeling assistance, and (4) allow hierarchical annotations. We note that annotation interfaces are typically independent of databases for storing and working with the resulting data.

### Data augmentation

Annotation efforts of any kind are expensive in terms of both human labor and monetary cost, limiting the availability of training data. Data augmentation is a common procedure to subtly alter the appearance of labeled ROIs—via geometric transformations or filtering operations—to avoid overfitting the model. Done appropriately, the approach can improve the representational power of the automated system. There is also potential for producing synthetic training data via 3D models or image merging before training (Mahmood et al. 2020). All these operations should be undertaken conservatively and in consideration of the target trait to avoid overfitting one's model to the augmented data.

### Datasets for transfer learning and feature extraction

Transfer learning is an effective way to reduce the initial annotation effort when preparing data to train a deep neural network architecture for a new task. The parameters of a model originally trained on a generic dataset like ImageNet are fine‐tuned to the new target dataset (Yosinski et al. 2014). The method has been used for classification tasks for plankton data (Orenstein and Beijbom 2017; Cheng et al. 2019). There are currently three large, publicly available plankton image datasets sorted for classification tasks that can be leveraged for such designs (Sosik et al. 2014; Cowen et al. 2015; Elineau et al. 2018).

Transfer learning is also an effective approach for repurposing trained networks for other computer vision tasks such as object detection or semantic segmentation (Girshick et al. 2014; Long et al. 2015; Ouyang et al. 2016). Doing so for plankton images will require generating sufficiently large labeled datasets akin to those produced for classification tasks. Codifying the annotation format across imaging systems—and making the resulting datasets public—will speed development of effective systems. Moreover, if these datasets are made public, consistent data formats will ease interoperability and speed development.

Feature extraction from deep nets—using internal representations of a model—is another viable option for rapidly generating features and classifiers from previously trained generic classifiers (Donahue et al. 2014). These weights can be used for unsupervised clustering or to train ensemble or margin classifiers (González et al. 2019; Schröder et al. 2020).

## Algorithms, approaches, and evaluation

**Fig. 4:**
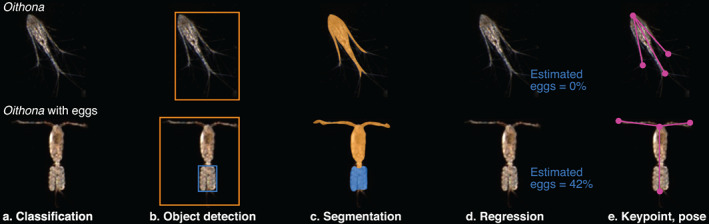
Examples of several techniques for trait extraction from zooplankton images. The hypothetical use case is examining ovigerous copepods imaged by the Scripps Plankton Camera system. The top panel is a non‐egg bearing copepod. The bottom panel is an individual carrying an egg‐sac. (**a**) Automated classifiers could be trained to add a semantic descriptor to the taxonomic class. (**b**) Object detection finds the organism and desired trait. (**c**) Segmentation algorithms classify the pixels as belonging to the organism or the trait. (**d**) Regression estimates the percentage of pixels that represent the trait. (**e**) Keypoint/pose estimation finds body nodes (red dots) and connects them (yellow lines) to estimate orientation or appendage extension.

**Fig. 5:**
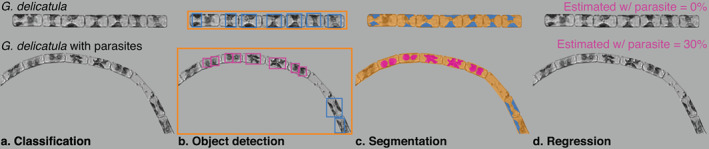
Examples of several techniques for trait extraction from phytoplankton. The hypothetical use case is examining parasitized diatom chains imaged by the Imaging FlowCytobot. The top panel is a healthy *Guinardia delicatula*. The bottom panel is a parasitized chain. (**a**) Automated classifiers could be trained to add a semantic descriptor to the taxonomic class. (**b**) Object detection finds the entire chain, chloroplasts, and parasites. (**c**) Segmentation algorithms classify individual pixels as belonging to the organism, chloroplasts, or parasites. (**d**) Regression estimates the amount of an image that corresponds to the organelle/parasite biovolume.

There are a myriad of algorithms and approaches that have been developed for computer vision tasks that could be adapted for trait‐based projects. As motivating examples, consider two hypothetical studies: one examining ovigerous copepods (Fig. 4) and another looking at parasitized diatoms (Fig. 5; Peacock et al. 2014). Note how distinct these cases are: the target organisms differ an order of magnitude in size, are imaged with very different instruments, and occupy different ecological niches. We highlight several broad categories of techniques that might be used to develop a pipeline to generate the required data: handcrafted features, semantic classification, object detection, segmentation, deep regression, and keypoint estimation. We note there are many specific algorithms, training regimes, hyperparameter tuning procedures, and, in the case of deep learning, loss functions within each category that can greatly impact system performance. Here, we seek to give an overview of each area without making specific recommendations as to the exact approach to pursue; it is not an exhaustive implementation guide. Most of the following subsections focus on the potential application of deep‐learning models, rather than those based on human‐engineered features, in keeping with the broad thrust of research in the computer vision community; modern representation learning techniques tend to outperform earlier approaches.

### Handcrafted features and measurable traits

Morphological traits measurements can be made by extracting hand‐engineered features from ROIs. Such measurements are made directly from the pixels and might include major axis length, ESD, or opacity. Techniques for doing so are well‐established and standard in many in situ imaging pipelines (Blaschko et al. 2005; Sosik and Olson 2007; Gorsky et al. 2010). If the instrument produces color images, the color channels could be used to infer information regarding an organism's pigment content or transparency. Calibrating these approaches can be done concisely by comparing automated measures with ground truth measurements made by a human operator.

Morphological traits measurements (in the biological sense) are the most directly interpretable, but require careful calibration of the pixel size and sample volume of a particular instrument. Likewise, interoperability can be challenging since the feature extraction technique will need to be rewritten for a new instrument—there is no general use feature extractor for different systems. However, once measurements are made and converted into realistic units, the metrics are easily comparable across devices.

### Classification with trait‐specific classes

Automated classifiers can be retrained to target specific traits with a semantically, rather than taxonomically, labeled set of images. If one wishes to separate ovigerous copepods from other copepods, they could add a training category specifying the semantic class “with eggs” (Fig. 4a). Similar semantic categories could be constructed to address traits such as body extension, presence of parasites, or behavioral signatures (e.g., extended appendages, body position, etc.). Any classification method could be used in this manner: ensemble, margin, deep nets, or otherwise. This has already been done to some extent in the publicly available IFCB and ISIIS datasets (Sosik et al. 2014; Cowen et al. 2015). Both datasets currently contain a number of classes distinguished with semantic descriptors related to traits: “G_delicatula_parasite” or “G_delicatula_external_parasite” from the IFCB (Fig. 5a) and “trichodesmium_puff” or “trichodesmium_tuft” from the ISIIS. Several classification efforts on these and other datasets already include such trait information (Luo et al. 2018; Ellen et al. 2019; González et al. 2019).

These efforts can be expanded in a targeted way by labeling (or re‐labeling) images with trait information and training a new model. The resulting classifier could then be run on new unlabeled data or as a second stage analyzer applied after initial classification. Evaluating the output could be done with raw accuracy on a per class basis or as the mAP (Table 2). As with any automated classification pipeline, care will need to be taken to assess issues related to dataset shift (Moreno‐Torres et al. 2012; González et al. 2017).

### Object detection for trait identification

Object detection pipelines are built to identify, select, and propose labels for subregions in an image (Zhao et al. 2019). Such techniques could be used to select egg sacs from the rest of a copepod's body or parasites inside a diatom chain (Figs. 4b, 5b). Other target traits might be bioluminescent structures, lipid reserves, or specific body parts. The models could also be used to locate organisms in a full frame image (Katija et al. 2021). Training object detection models for such tasks will require new training sets that include bounding box coordinates and labels targeting the traits. The resulting bounding boxes could be used for count‐based studies like estimating the number of ovigerous individuals or the percentage of a population that has been feeding.

Object detection was historically accomplished by proposing regions based on hand‐engineered features from an input image and a localizer searching the feature space (Lienhart and Maydt 2002; Viola and Jones 2004; Felzenszwalb et al. 2010). Recently, deep neural networks—such as Faster Regions with Convolutional Neural Network (CNN) Features (RCNN), Single Shot Multibox Detector (SSD), and You Only Look Once—have been specifically designed to identify and select subregions of images (Girshick et al. 2014; Redmon et al. 2016; Liu et al. 2016a). These techniques are generally used to draw bounding boxes around objects in natural image datasets like COCO and VOC. To use these methods, aquatic ecologists will need to localize traits with bounding boxes stored in a consistent format encoding their location and size. Object detectors are typically evaluated with the mean mAP score (Table 2).

### Semantic and instance segmentation

Instead of selecting bounding boxes around subregions, segmentation algorithms attempt to delineate sections of the image at the pixel level. Rather than returning boxes surrounding interesting areas of an image, segmenters produce masks where each pixel is tagged as belonging to a particular region. Segmentation approaches are appropriate for trait studies that require estimating mass. A computer could be trained, for example, to select pixels that represent pigmented regions of an organism, gut content, the egg tissue, or parasites (Figs. 4c, 5c). The resulting masks could then be used to estimate an organism's state (healthy/starved, ovigerous/not) or the proportion of tissue dedicated to such functions.

Preparing data for training segmentation networks will require very detailed human delineated, pixel‐level masks. The annotation task thus requires more time and attention from the human expert than object detection or classification labeling. Superpixel‐based assistance workflows can be used to accelerate the annotation task (King et al. 2018). Segmentation tasks are also typically more computationally difficult than detection. There are again many flavors of deep segmentation networks. At the time of writing, UNet, Segnet, and Mask RCNN are among the most widely applied models for generic object segmentation tasks (Ronneberger et al. 2015; Badrinarayanan et al. 2017; He et al. 2017). The performance of segmentation algorithms of all types is typically stated as the AP at different levels of intersection over union (Table 2).

The computer vision community makes a distinction between “semantic” segmentation—labeling all pixels with a class—and “instance” segmentation—separating each occurrence of a given class. For example, consider an image of two touching copepods (Fig. 2u): semantic segmentation would label all “copepod” pixels as a single region while instance segmentation would label them as “copepod 1” and “copepod 2.”

### Deep regression

The ultimate goal of any of the techniques we have mentioned is to output an estimate of some relevant trait—angle relative to horizontal, extension of limbs, presence of eggs, etc. Rather than learning an intermediate task like segmentation or object detection, deep regression algorithms seek to train a model to directly output these estimates. Regression approaches are thus appropriate for calculating numerical properties of an object. Returning to the copepod egg example, rather than training an object detector or segmenter, an operator could instead build a network that outputs an estimate of the percentage of the ROI occupied by eggs (Fig. 4d). Likewise, a deep regressor could be trained to estimate the percentage of a chain ROI that is parasitized (Fig. 5d).

Deep regression approaches have been successfully implemented on photos of humans to directly estimate head pose and a subject's age (Liu et al. 2016b; Rothe et al. 2018). Using deep regression in this manner will discard other information in the image, obviating the need for computationally complex object detection and segmentation, potentially yielding a more efficient means of getting the value of a trait that can be expressed as a continuous number (Lathuilière et al. 2020). Scientists wishing to use deep regression will need to associate each image in their training set with a number (e.g., percent egg mass or amount of parasitized tissue; Figs. 4d, 5d) representing the target trait. The efficacy of these techniques can be expressed as the mean squared error of the predicted estimate.

### Pose or keypoint estimation

Pose estimation networks attempt to teach computers to recognize how a body is positioned in an image (Sapp and Taskar 2013; Andriluka et al. 2014). These algorithms search for and connect keypoints that together describe a very rough skeleton. Keypoint analysis is ideal for studying organism orientation and limb extension. By identifying head, tail, and extremities, the computer can estimate what direction the organism is facing relative to the camera and the angle of its body parts (Fig. 4e). Such an approach could also be used to investigate interactions between organisms from full frame images.

Currently, pose estimators are almost exclusively used to find people and determine their 3D orientation. Several recent papers have suggested adapting these methods to animal images both in the wild and the lab (Cao et al. 2019; Li et al. 2020). Keypoint and pose tasks are relatively new as compared to object detection and segmentation. A few contemporary approaches are PersonLab, OpenPose, and DensePose (Cao et al. 2017; Papandreou et al. 2017; Alp Güler et al. 2018). Keypoints are typically evaluated by specifying a threshold distance from the true point location. Point proposals can then be evaluated with accuracy as a function of the threshold. Developing training data for such tasks requires researchers to select relevant points in an ROI and save their coordinates as *xy* pairs.

## Evaluation metrics

There are many evaluation metrics used to assess the efficacy of ML systems, and several that are particularly important to the methods we have discussed (Table 2; Supporting Information S1). Selection of the appropriate metric can better guide training and deployment of ML systems by providing informative feedback on the system performance. Classifier accuracy, the average percent of correctly returned labels over all classes, is the most commonly reported metric for plankton classification systems. Accuracy is a useful but flawed metric, often obscuring important errors since it only states the rate of correct labels (Tharwat 2020). Practitioners should rather rely on more informative metrics like precision, recall, and the F1‐score that explicitly encode false positives and negatives between classes (Table 2; Supporting Information S1).

Object detectors and segmentation algorithms are often evaluated with the mAP score, as defined in the VOC and COCO challenges. The number encapsulates the trade‐off between precision at different levels of recall or Intersection over Union (Supporting Information S1). The mAP score is the standard evaluation metric for evaluating these models and are likewise critical to evaluate such procedures for ecological applications. Deep regression and keypoint estimation might use more familiar metrics like mean average or absolute error.

Time and energy should be put into selecting and understanding these metrics, as they will guide model development and deployment. These metrics can allow savvy observers to diagnose biases in their training data and assess the efficacy of a model. In an evaluation‐first design scheme these insights can allow researchers to quickly revise their strategy by altering human annotation goals or selecting alternative training approaches (Fig. 3).

## Perspectives and conclusion

### Using ML for trait estimation: A promising tool

Digital imaging systems coupled with automated analysis techniques are increasingly used by aquatic ecologists. Much of the community's effort has been focused on developing classification approaches, largely delineated along taxonomic lines (Irisson et al. 2022). We believe that the extraction of functional traits is the next evolution in ML‐enabled analysis of ecological images. With traits values derived from in situ images, aquatic ecologists could automatically estimate inter‐ and intraspecific variability of functional traits, better link diversity to ecosystem functioning, and parameterize and test trait‐based models to assess the health of aquatic ecosystems (Martini et al. 2021).

As with classification efforts, developing trait extraction systems will require a substantial investment of expert human analysis. Images will need to be closely scrutinized and appropriately labeled, sometimes at the pixel level, to perform experiments and validate new approaches. To this end, we strongly advocate researchers employ the evaluation‐first design paradigm, where the target trait and criteria for success are chosen before any annotation begins. Doing so will ensure that labels are stored in a format compatible with a desired model and enough data are annotated for the experiments, thus facilitating interoperability.

All of the computational approaches presented have the potential to yield significant new scientific results by revealing individual level trait metrics across a population. We believe that deep regression and pose estimation are especially promising for plankton trait studies. Pose estimation could be used to examine the orientation of a group of organisms relative to horizontal or the angle of their appendages relative to their body. Deep regression would be effective for estimating proportion in an image: for wxample, how extended are the appendages or mucus net, how full is the gut, how big is the lipid reserve, how curved or deformed is the shell, etc. This approach will require segmentation style annotations but is ultimately less computationally intensive to train and deploy. We stress that this paper is meant to direct readers to broad sets of ML tools; there are a multitude of viable combinations of traits, ML techniques, and statistical analyses. Moreover, new computer vision approaches are being released every year; many of the specific models mentioned in this paper will very likely be obsolete within a few years.

We anticipate that FTBAs via imaging will be maximally effective when undertaken with a suite of instruments targeting overlapping portions of the planktonic size spectrum. Facilitating studies at such a broad scale requires conscientious calibration of pixel size and sample volume for all instruments capturing images of plankton. Indeed, calibration is arguably more important for trait extraction than for taxonomic classification. At a minimum, the pixel size has to be well‐established and reported to ensure that recovered measurements are appropriately scaled. While the estimation of pixel size is possible for microscopic imaging systems that are imaging precisely in a single plane, accurate pixel size calibration cannot be achieved with single cameras; stereo vision or active illumination is required to recover the scale. Ideally all sources of variability, such as intensity and blur characteristics, will be quantified and made widely available (Vandromme et al. 2012; Giering et al. 2020). While sharing these parameters will aid cross‐dataset comparisons, true interoperability between in situ imaging tools will be achieved only through extensive intercalibration studies.

### From individual traits to ecosystem functioning

The computer vision and ML techniques we discussed will yield previously inaccessible data: consistent, large‐scale quantitative measures of trait expression at the individual level. With these measurements, single trait distributions can be estimated in space and time (Barton et al. 2013; Brun et al. 2016; Carmona et al. 2016) and intraspecific variability of traits could be documented (Martini et al. 2021). Statistical models can subsequently be implemented to better understand the interplay between trait expression and environmental factors by deriving empirical relationships between them. These empirical relationships could further be used to structure and parameterize mechanistic models of planktonic communities based on equations describing functional types and traits (Follows et al. 2007; Stemmann and Boss 2012; Ward et al. 2012; Le Quéré et al. 2016; Serra‐Pompei et al. 2020). Marrying large‐scale observational capacity and modeling in this manner would allow scientists to examine evolutionary constraints on trait expression at unprecedented spatiotemporal scales.

The estimation of functional traits at the individual level will also yield further knowledge on ecosystem functioning by giving access to bulk trait properties at the population or community levels, including community‐scale insight into the ecology of patchiness, individual interactions, and associated advantages for resource acquisition such as light and nutrients (Litchman and Klausmeier 2008). For instance, sex ratio could also be estimated from images, since male and female zooplankton are often distinguished by traits that affect physiology, behavior, and eventual fitness (Heuschele et al. 2013). Females typically invest more resources in gonad development and offspring production, often incurring higher predation risks as a trade‐off. For example, during DVM, females of the krill species *Meganyctiphanes norvegica* migrate significantly closer to the surface to forage at night than males (Tarling 2003). This behavior leads to 40% higher energy intake, yet increases the risk of visual predation, resulting in the male‐to‐female sex ratio shifting from 1 : 1 at the beginning of the summer to 3 : 1 by the onset of winter. The ability to record finely resolved vertical profiles of sex‐associated trait frequencies could allow direct quantification of mating seasonality and potential mating success and output. Such observations could also allow detecting potential ecological perturbations as deviations from a baseline distribution of traits. At the community scale, image‐based functional traits, such as size, photosynthetic traits, metabolic rates, or vertical migration, provide further access to ecosystem functioning through the estimation of primary production (Litchman et al. 2015) or carbon export (Stamieszkin et al. 2015; Archibald et al. 2019; Kiko et al. 2020).

### Desirable qualities for future instruments

Future development of image acquisition systems should consider trait analysis in the hardware and software design phase. Four particular capabilities would be informative for trait‐based studies: (1) the ability to record color images, (2) the capacity to record bursts of video, (3) the storage to retain full‐frame images (or the necessary metadata to reconstruct them from ROIs), and (4) 3D imaging capabilities. Color information, either as standard 3‐channel color or hyperspectral, will facilitate studies seeking to quantify pigmentation, gut content, or bioluminescence with both segmentation and object detection approaches. Recording video will make it easier to assess interactions between individuals with real‐time object detectors. Retaining full frame images would allow revisiting them and detecting objects that were not targets during the initial processing. Increased storage capacity and speed will allow instruments to hold more data, such as videos and full frames, while maintaining deployment duration. 3D imaging via stereo imaging, structure from motion, or Z‐stack image acquisition would provide better descriptions of organism volume and other structures with higher dimensional segmentation models.

All of these new modalities are rapidly becoming realistic for long‐endurance, autonomous plankton imaging systems. Disk space is continually becoming less expensive, bus rates are getting faster, and embedded computers are getting more power efficient. While the computer components are available, the actual construction and deployment will necessitate significant development time and effort. In particular, given enough power, on‐board processing techniques could be explored to automatically detect possible interactions or other potentially interesting events to trigger video recording.

### Beyond plankton

Plankton are far from the only organisms sampled by imaging systems. Indeed, ecologists collect untold exobytes of image and video data, including other marine organisms such as fish, benthic organisms (Beyan and Browman 2020), mammals (O'Connell et al. 2010; Karnowski et al. 2016), and freshwater benthic organisms (Milošević et al. 2020). Research teams that produce such data have already begun to leverage ML techniques to analyze their data, largely relying on object detection and taxonomic classification approaches (Allken et al. 2019; Kloster et al. 2020; Mahmood et al. 2020). There remains much to be learned by studying these data streams with an eye toward trait‐based analyses. So far, only a few studies have quantified traits automatically and have generally focused on size estimates (Álvarez‐Ellacuría et al. 2020).

The ML and computer vision methods we have identified could be adapted to estimate the number of parasites per fish in salmon farms (via object detection), the bleaching of coral reefs (via segmentation or deep regression; Nielsen et al. 2018), or the cell deformations of benthic diatoms in response to pollutants (with keypoint estimation or deep regression). Conversely, plankton studies could benefit from approaches developed to automatically analyze images of other aquatic organisms (e.g., Beyan and Browman 2020 and references therein). Scientists studying benthic macroorganisms, fish, or marine mammals with images will have unique sets of technological challenges associated with automating analysis (e.g., complex backgrounds for in situ benthic images or overlapping targets when recording pelagic fish). Despite such difficulties, functional traits could still be estimated from images and feed multicompartment studies (Martini et al. 2021). The eventual combination of image datasets to cover the wide range of organism size and habitat will likely require both careful intercalibration and new mathematical methods.

### Conclusion

As a final note, we would like to advocate for two goals that we should pursue as a community: (1) more open and efficient sharing of trait‐annotated datasets, and (2) development of educational programs at the interface of computer science and ecology. The first goal is obvious and one that has been discussed widely. The benefits of open‐access annotations are manifest: combining large volumes of publicly available labeled data with fine‐tuning procedures could speed testing and deployment of new techniques. For instance, the Kaggle challenge on plankton image classification (https://kaggle.com/c/datasciencebowl/) has promoted the use of CNNs. This stressed how beneficial data sharing can be, since the problems that ecologists are facing with their images (e.g., taking object size into account or dealing with unbalanced datasets) can be challenging for data scientists as well. Therefore, aquatic ecologists should be encouraged to share or continue sharing their image data and models. The second goal will require more time and effort, but will likewise benefit the field as a whole. Most universities do not offer interdisciplinary tracks for students to pursue ML for ecological data. The establishment of dedicated programs that combine computer vision, ML, and aquatic ecology training should be promoted. The goal of such initiatives should not be to make ecologists into ML experts nor vice versa; there are too many complexities inherent in both fields for that to be a reasonable expectation. Instead, such educational initiatives should seek to ease the impedance mismatch between practitioners in both areas. Offering degrees and certificates at this interface would better prepare future scientists, establish a talent pool of creative practitioners, and encourage the sustained dialogue necessary to build fruitful collaborations at this rich interdisciplinary juncture.

## Conflict of Interest

None declared.

## Supporting information


**Appendix S1** Supporting Information

## Endnotes

^1^
Note that in this paper “morphology” should be understood in its biological sense, that is, the visually identifiable properties of an object, rather than in its computer vision sense, that is, the numerical characteristics derived from the binary mask of the object (its “imprint” in the image).

^2^

http://host.robots.ox.ac.uk/pascal/VOC/voc2007/htmldoc/index.html

^3^

https://cocodataset.org/#format-data
