# Supplementary material for: Machine learning techniques to characterize functional traits of plankton from image data
Source: Limnol Oceanogr. 2022 Jun 30;67(8):1647–69. doi: 10.1002/lno.12101 (PMC9543351; doi:10.1002/lno.12101)
Supplement: Supplementary file 1 — Appendix S1 Supporting Information [file LNO-67-1647-s001.docx]

# Supplemental Information: Machine learning techniques to characterize functional traits of plankton from image data

Eric C. Orenstein^1^, Sakina-Dorothée Ayata^1,2^, Frédéric Maps^3,4^, Tristan Biard^5^, Érica C. Becker^6^, Fabio Benedetti^7^, Thibault de Garidel-Thoron^8^, Jeffrey S. Ellen^9^, Filippo Ferrario^3,4,10^, Sarah L.C. Giering^11^, Tamar Guy-Haim^10^, Laura Hoebeke^13^, Morten Hvitfeldt Iversen^14^, Thomas Kiørboe^15^, Jean-François Lalonde^16^, Arancha Lana^17^, Martin Laviale^18^, Fabien Lombard^1^, Tom Lorimer^19^, Séverine Martini^20^, Albin Meyer^18^, Klas Ove Möller^21^, Barbara Niehoff^14^, Mark D. Ohman^9^, Cédric Pradalier^22^, Jean-Baptiste Romagnan^23^, Simon-Martin Schröder^24^, Virginie Sonnet^25^, Heidi M. Sosik^26^, Lars S. Stemmann^1^, Michiel Stock^13^, Tuba Terbiyik-Kurt^27^, Nerea Valcárcel-Pérez^28^, Laure Vilgrain^1^, Guillaume Wacquet^29^, Anya M. Waite^30^, Jean-Olivier Irisson^1^

Supplemental Information 1: Evaluation Metrics

Accuracy ($acc$) is by far the most widely used and intuitive evaluation metric for classification problems. In the binary case, it is simply the number of correctly returned labels in the “positive class” over all classified data points. For multiclass systems, $acc$ can be computed as the sum of the diagonals of the confusion matrix divided by the total number of objects classified. In both cases $acc$ ranges from 0 to 1 with values closer to 0 indicating poor performance and closer to 1 suggesting near perfect performance. $acc$ provides a useful snapshot of classifier performance, but obscures information regarding the source of errors and is very sensitive to class distribution (Tharwat 2020).

Precision ($p$) and Recall ($r$) are related to $acc$ and similarly reflect a classifier’s performance. $p$ reflects how accurate the predictions are, while $r$ describes the classifiers ability to retrieve relevant data points. These two metrics are tightly coupled: when a system's precision increases, its recall decreases since it returns more of the possible true labels at the expense of more false positives.

Average Precision ($AP$) summarizes the trade off between the precision and recall for a given class by averaging the precision for different levels of the recall. Computing AP requires the algorithm to return either probability of class membership or confidence values rather than hard class assignments (Everingham et al. 2010; Hoiem et al. 2012). The better the model is at prediction, the higher the precision at all values of recall thus increasing $AP$. $AP$ ranges between 0 and 1, with 1 being a perfect model that returns all relevant objects with high confidence. $AP$ is computed on a class-by-class basis and can be summarized for a whole model by averaging over all the classes. The mean Average Precision ($mAP$) encapsulates the system's precision and recall over all classes in the space.

Object detection and segmentation algorithms seek to both localize and describe regions in an image. Metrics such as $AP$ can be adapted to evaluate how well such systems localize a particular class. For the COCO benchmark dataset, for example, results are typically stated as $AP$ as a function of Intersection over Union ($IoU$) thresholds (Lin et al. 2014; He et al. 2017). $IoU$ is a pixel level computation of the ratio between the area shared correctly by the computer and the total number of pixels between the proposal and the ground truth. If the proposed region and the ground truth are perfectly overlapping, $IoU=1$. If they are not intersecting at all, the $IoU\simeq0$ (Rezatofighi et al. 2019).

References

Everingham, M., L. Van Gool, C. K. Williams, J. Winn, and A. Zisserman. 2010. The PASCAL Visual Object Classes (VOC) challenge. Int. J. Comput. Vis. **88**: 303–338.

He, K., G. Gkioxari, P. Dollar, and R. Girshick. 2017. Mask R-CNN. Proceedings of the Proceedings of the IEEE International Conference on Computer Vision. 2961–2969.

Hoiem, D., Y. Chodpathumwan, and Q. Dai. 2012. Diagnosing error in object detectors. *European Conference on Computer Vision*. Springer. 340–353.

Lin, T.-Y., M. Maire, S. Belongie, J. Hays, P. Perona, D. Ramanan, P. Dollár, and C. L. Zitnick. 2014. Microsoft COCO: Common Objects in COntext. *European Conference on Computer Vision*. Springer. 740–755.

Rezatofighi, H., N. Tsoi, J. Gwak, A. Sadeghian, I. Reid, and S. Savarese. 2019. Generalized intersection over union: A metric and a loss for bounding box regression. *Proceedings of the IEEE Conference on Computer Vision and Pattern Recognition*. 658–666.

Tharwat, A. 2020. Classification assessment methods. Appl. Comput. Inform. **17**: 168-192. doi:10.1016/j.aci.2018.08.003
